# Anthropomorphic or non-anthropomorphic? Effects of biological sex in observation of actions in a digital human model and a gantry robot model

**DOI:** 10.3389/fnbot.2022.937452

**Published:** 2022-08-17

**Authors:** Miriam Abel, Sinem Kuz, Harshal Jayeshkumar Patel, Henning Petruck, Juliane Klann, Christopher M. Schlick, André Schüppen, Antonello Pellicano, Ferdinand C. Binkofski

**Affiliations:** ^1^Division for Clinical and Cognitive Sciences, Department of Neurology Medical Faculty, RWTH Aachen University, Aachen, Germany; ^2^Speech-Language Pathology, Department of Rehabilitation and Special Education, Faculty of Human Sciences, University of Cologne, Cologne, Germany; ^3^Institute of Industrial Engineering and Ergonomics, RWTH Aachen University, Aachen, Germany; ^4^SRH University of Applied Health Sciences, Heidelberg, Germany; ^5^Interdisciplinary Center for Clinical Research – Brain Imaging Facility, University Hospital Aachen, Aachen, Germany; ^6^Institute of Neuroscience and Medicine (INM-4), Research Center Jülich GmbH, Jülich, Germany

**Keywords:** anthropomorphism, action observation system, gender effect, human-robot interaction, motion perception, digital human model, gantry robot model

## Abstract

Robots are ever more relevant for everyday life, such as healthcare or rehabilitation, as well as for modern industrial environment. One important issue in this context is the way we perceive robots and their actions. From our previous study, evidence exists that sex can affect the way people perceive certain robot's actions. In our fMRI study, we analyzed brain activations of female and male participants, while they observed anthropomorphic and robotic movements performed by a human or a robot model. While lying in the scanner, participants rated the perceived level of anthropomorphic and robotic likeness of movements in the two models. The observation of the human model and the anthropomorphic movements similarly activated the biological motion coding areas in posterior temporal and parietal areas. The observation of the robot model activated predominantly areas of the ventral stream, whereas the observation of robotic movements activated predominantly the primary and higher order motor areas. To note, this later activation originated mainly from female participants, whereas male participants activated, in both robot model and robotic movements contrasts, areas in the posterior parietal cortex. Accordingly, the general contrast of sex suggests that men tend to use the ventro-dorsal stream most plausibly to rely on available previous knowledge to analyze the movements, whereas female participants use the dorso-dorsal and the ventral streams to analyze online the differences between the movement types and between the different models. The study is a first step toward the understanding of sex differences in the processing of anthropomorphic and robotic movements.

## Introduction

**Figure 1:**
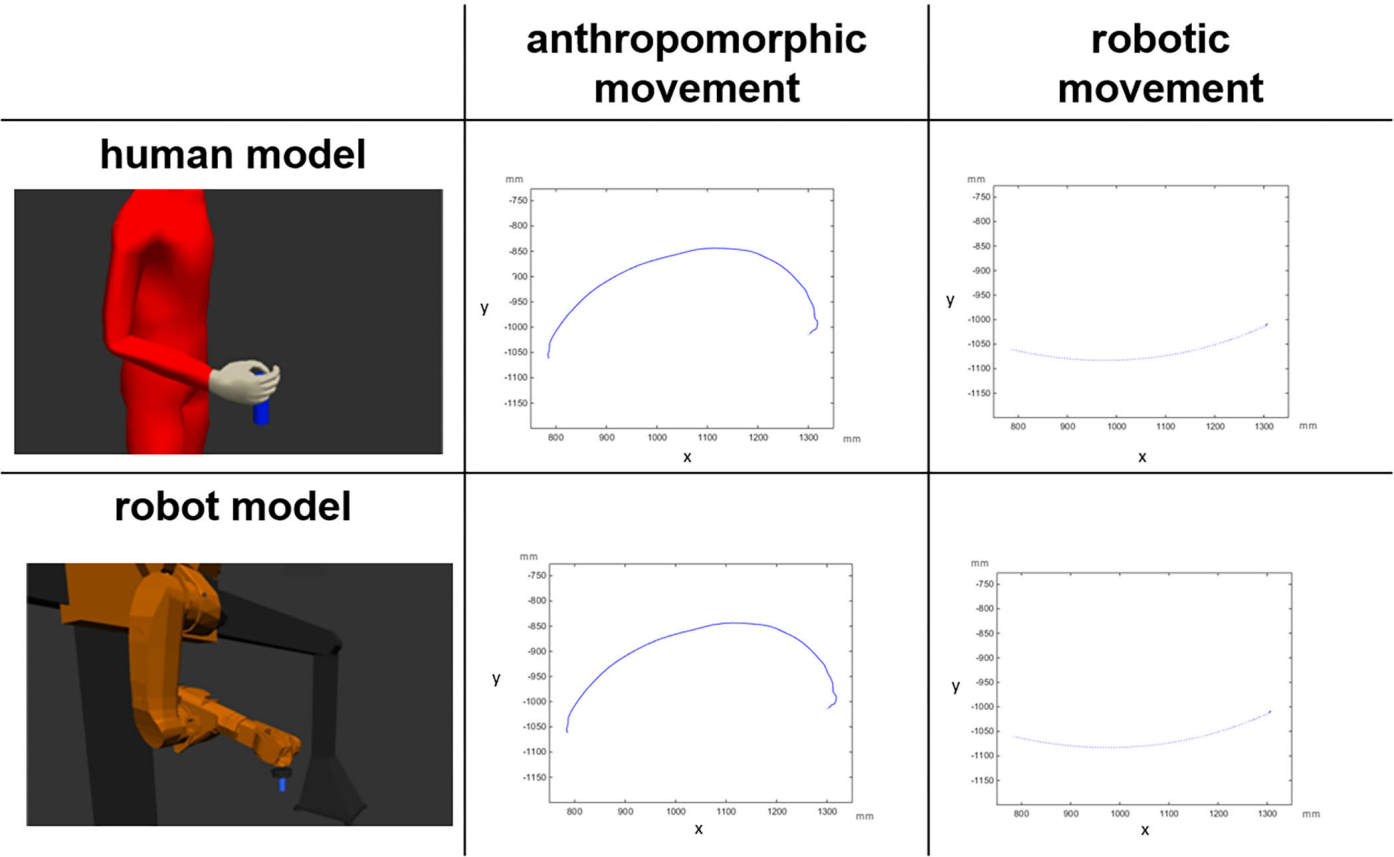
Visual representation of the factorial design: the depicted trajectories (projections on the X,Y plane) belong to a digital human model and to a gantry robot model performing anthropomorphic and robotic movements. The unit is millimeter (mm) (Abel et al., 2020).

The presence of robots in our everyday life is increasing steadily. The integration of robots as co-workers is a common practice in modern industrial production, healthcare industry, and rehabilitation (Karwowski, 1991; Michalos et al., 2022). Michalos et al. (2022) report about the different approaches on the implementation of human robot collaborative applications. In particular, during the COVID-19 pandemic, robots were used as humanoid service robots (Ozturkcan and Merdin-Uygur, 2021). To regulate stress, strain, comfort, and trust during human-robot interaction, robotic co-workers must meet safety standards, especially the identification with a robot as a co-worker is based on the attitude toward a robot, technological expertise, and personality (Savela et al., 2021). A sense of comfort in the workplace is essential and may be achieved through the discussion of several relevant factors. The prerequisite of safe and flexible interaction with robot partners is the interpretation of their movements. Independent of the shape of a robot, its movements can be classified as *robotic*, point to point movements (Figure 1), and *anthropomorphic* movements, that is, humanlike movements for which observers attribute human traits to non-human entities (Epley et al., 2007; Złotowski et al., 2017).

In human-robot interaction (HRI), there is a large debate on the role of sex^1^ differences in operators on the perception of robots. Lee (2008) reported that women showed more positive reactions than men when interacting with a flattering robot with a human voice, but no differences were observed when the robot was assigned a machine voice. In a group of children, Cameron et al. (2018) found that male participants who interacted with a responsive facially expressive robot, showed a positive affective response and indicated greater liking toward the robot, compared with male participants who interacted with the same robot with a neutral expression. Female participants, instead, showed no marked difference across the conditions. In line with these findings, Stafford et al. (2014) found, in a group of elderly participants, a better robot attitude for male than for female participants. These results suggest the existence of a bias for male participants interacting with robots that is independent of age.

More specifically, Abel et al. (2020) investigated sex differences in the perception of robotic and anthropomorphic movements. In this study, a digital human model and a virtual gantry robot model performed anthropomorphic movements mapped from human kinematics, and robotic, point-to-point movements. Two groups of male and female participants rated the perceived level of anthropomorphism of each of the four model and movement combination. The human model was not perceived as more anthropomorphic than the robot model in both male and female groups. However, male participants rated anthropomorphic movements more anthropomorphic than robotic movements, whereas female participants rated the two movements equally. To complete the picture, male participants rated the robotic movements as less anthropomorphic than female participants did, while their rating of the anthropomorphic movements did not differ from the female rating. Thus, men were more sensitive than women in terms of differences between robotic and anthropomorphic movements, but women overall experienced movements as more anthropomorphic than men.

Studies have investigated the neural underpinnings of human-robot interaction. Cross et al. (2012) demonstrated that, independent of the perceived human or robot agent, the *action observation network* (i.e., the brain circuit that is activated when observing other people in action, which consists of premotor, parietal, and occipito-temporal areas) showed higher activation during the observation of robotic motion cues than of natural human motion cues. The authors concluded that the action observation network is not involved in familiar observed action. Similar findings were described in previous studies in which activation in the parietal node of the action observation network were reported when robots performed robotic goal-directed movements (Hamilton and Grafton, 2008; Ramsey and Hamilton, 2010). Furthermore, Liepelt et al. (2009), Liepelt and Brass (2010), and Liepelt et al. (2010) supported the involvement of the action observation system in goal-directed and contextual familiar movements, with a bias to animated human agents. In contrast, Gazzola et al. (2007) found no difference in the activations during observation of anthropomorphic and robotic actions. Also, Chaminade et al. (2010) described no differences in the activation of the action observation system during the observation of robot and human agents. These results (see also Urgen, 2015; Hoenen et al., 2016) may be explained by the fact that the movements performed by human and robot models were not aimed at a goal.

Another line of research focuses on early stages of visual processing (Giese and Poggio, 2003; Blake and Shiffrar, 2007). The processing of actions in the early visual cortex needs two important visual cues, namely, form and motion of the actor (Urgen et al., 2019). Referring to this, two parallel pathways of the visual system have been discussed (Mishkin and Ungerleider, 1982). The *dorsal stream*, that is associated with motion information, and the *ventral stream* that processes form information (Cross et al., 2016; Urgen et al., 2019; Urgen and Saygin, 2020). The dorsal stream is also called “how” system (Goodale and Milner, 1992) or “where” system (Ungerleider and Mishkin, 1982) regarding the function of localizing objects in visual space, whereas the ventral stream is referred to as “what” system, and plays a role in the perceptual identification of objects and in the analysis of object characteristics (Ungerleider and Mishkin, 1982; Goodale and Milner, 1992; Milner and Goodale, 1995, 2008; Vry et al., 2009; Gallese, 2016). In more recent years, a further subdivision of the dorsal stream into a dorso-dorsal and a ventro-dorsal stream has been proposed (Rizzolatti and Matelli, 2003; Binkofski and Fink, 2005; Pisella et al., 2006; Binkofski and Buxbaum, 2013). The dorso-dorsal stream is responsible for online motor control with little working memory capacity and the ventro-dorsal stream is equipped with more working memory capacity and responsible for memory-driven motor control and motor simulation.

To date, there is an ongoing debate about sex differences in human-robot interaction. Previous studies investigated the influence of robots with different gender-specific markers on human's trust, interaction, and wellbeing toward robots (Bryant et al., 2020; Hover et al., 2021) as well as biological sex differences in humans in the interaction with robots (Reich-Stiebert and Eyssel, 2017; Beraldo et al., 2018). However, there are very few studies that investigated sex differences in brain functions that underpin behavioral differences in human-robot interaction. This study aimed at filling this gap. It was conceived as a direct functional magnetic resonance imaging (fMRI) follow-up of the behavioral study by Abel et al. (2020). Both behavioral and imaging data have been collected within the same experiment from the same group of participants. Indeed, we investigated the patterns of neural activation in male and female participants during the perception of anthropomorphic and robotic placing movements performed by two different models: (1) a virtual representation of a gantry robot and (2) a digital human model (Figure 1). We investigated the existence of differences between female and male humans in the brain processing of perceived anthropomorphic and robotic movements. Based on the behavioral results in Abel et al. (2020), we hypothesized that different sensitivities to anthropomorphic and robotic movements in male and female participants would correspond to different neural processing paths in the brain. Specifically, the two dorso-dorsal and ventro-dorsal processing streams should have a crucial role in the processing of perceived movements, in general, and in identifying sex differences, in particular.

## Materials and methods

### Participants

Imaging data were collected from the same group of participants as in the study by Abel et al. (2020). A total of 40 right-handed healthy volunteers, with twenty male and 20 female, participated in the study [20 female, mean age-−23.5 years (SD 5.9); 20 male, mean age-−24.8 years (SD 3.4)] after they gave their written informed consent. The study was approved by the Institutional Ethics Review Board of the Medical Faculty at RWTH Aachen University (EK 2013/14).

### Stimuli

The video stimuli were generated by the Chair and Institute of Industrial Engineering and Ergonomics, RWTH Aachen University (IAW). Two different models of robots were used: (1) an Editor for Manual Work Activities (EMA) (Fritzsche et al., 2011) to simulate the human model and (2) a virtual presentation of a gantry robot (Figure 1). Additionally, the IAW generated motion data for anthropomorphic and robotic (point-to-point) movements (Figure 1) (see Kuz et al., 2015 for further information about the generation of the motion data of the different videos).

All in all, we recorded eight movements for each human and robot model. Each model placed the cylinder on four different positions in a straight line. For the fMRI experiment, we selected three positions per model and per movement and presented 12 videos per block (three videos with anthropomorphic movements and three videos with robotic movements two times) with a total of eight blocks (four times human model and four times robot model). Each block had the same array: First, three anthropomorphic videos were presented with three different positions, followed by three robotic movements with three different positions. Subsequently, the same three anthropomorphic videos followed by the same three robotic videos were presented again, with the same arrays until a new block with another model started. The array of the blocks alternates between robot model and human model.

### Study design

The study features a factorial design with *sex* (male vs. female) as the between-participants factor, and with *model* (human vs. robot), and *movement type* (anthropomorphic vs. robotic) as the within-participants factors. Hence, we obtained four within-participant experimental conditions for each male and female group: human model performing anthropomorphic movements (HH), human model performing robotic movements (HR), robot model performing anthropomorphic movement (RH), and robot model performing robotic movements (RR, Figure 1).

### fMRI study design

**Figure 2:**
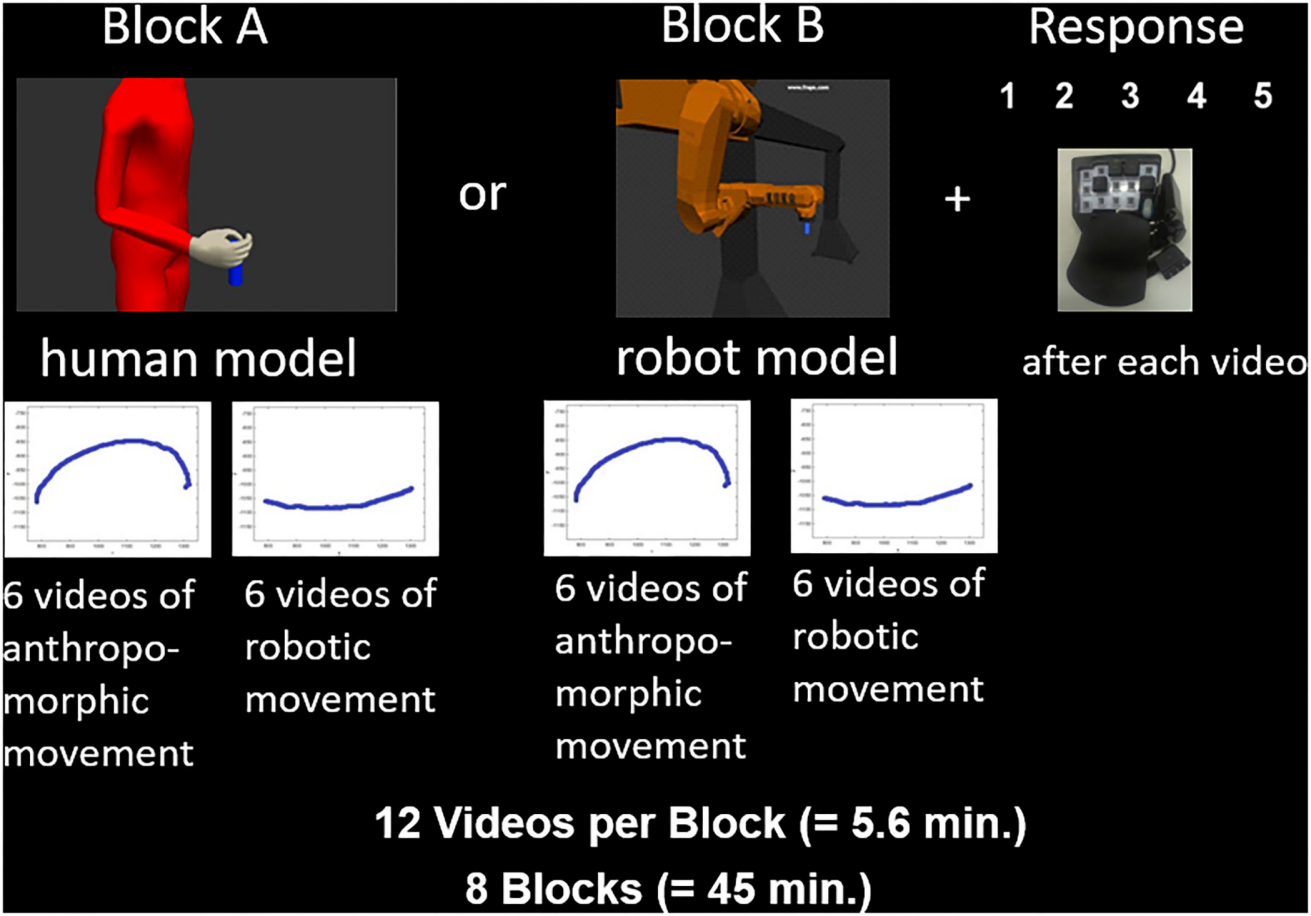
Study design of a fMRT trial.

First, the participants arrived and got some information about the study and the scanner. They gave their written informed consent and filled out a questionnaire to evaluate the inclusion criteria (sex, age, right handedness, normal or corrected-to-normal vision, absence of neurological or psychiatric diagnosis) and the career, the profession, and the highest degree of school education. Afterwards, the participants were introduced to their task in the scanner by giving an example of the task on the laptop and explaining shortly the differences of robotic (point-to-point) and anthropomorphic (humanlike) movements. Before lying in the scanner, the subject was instructed about the course of the experiment and the use of the answer box for judging the anthropomorphism of the movements. In the scanner, participants had the opportunity to perform some example exercises before the main experiment started. Here, each trial consisted of three steps. First, the participants were asked to watch the video and subsequently judge their perceived level of anthropomorphism of the model's movement. The videos were presented in full color with a resolution of 900 x 563 pixels using a back projection system, which incorporated an LCD screen placed behind the MRI scanner. A mirror installed above the participant's eyes provided a reflection of the screen. The participant was allotted 10 s to rate each model on a 5-point scale. Half of the participants used a scale from “very robotic” (score 1) to “very anthropomorphic” (score 5), and the other half from “very anthropomorphic” (score 1) to “very robotic” (score 5). To respond, participants used an fMRI-compatible response button box with three buttons to submit their ratings (to move left or right on the scale, and to confirm the answer). Each participant completed eight blocks with 12 trials. The conditions were counterbalanced across groups and participants; half of the participants started the experiment with the human model (Block A) and the other half with the robot model (Block B) (Figure 2).

A high-resolution T1-weighted anatomical scan was acquired with a 3T Siemens PRISMA MRI system using the 20-channel head coil (TR = 1,900 ms, voxel size = 1 × 1 × 1 mm^3^; TE = 2.21 ms; flip angle = 9°) for each participant. In order to minimize head motion artifacts, the participants' head position was stabilized using a vacuum pillow. Additionally, 12 functional imaging blocks sensitive to blood oxygenation level-dependent (BOLD) contrast were recorded for each participant (T2^*^-weighted echo-planar sequence, TR = 2,000 ms; TE= 30 ms; flip angle = 90°; voxel size = 3 × 3 × 3.6 mm^3^).

### Analysis of behavioral data

Mean anthropomorphism scores were submitted to a *sex* x *model* x *movement type* analysis of variance (ANOVA). For detailed information about the results, please refer to Abel et al. (2020).

### Analysis of imaging data

The anatomical scans were normalized and averaged in SPM 12 (http://www.fil.ion.ucl.ac.uk/spm/software/spm8/). The fMRI time series were corrected for movement. Images were than realigned to each participant's first image. Data were normalized into the standard MNI space. Images were resampled every 2.5 mm using fourth-degree spline interpolation and smoothed with a 9 mm FWHM Gaussian kernel to accommodate inter-subject variation in brain anatomy and to increase the signal to-noise ratio in the images. The data were high-pass filtered (128 s) to remove low-frequency signal drifts and corrected for autocorrelation assuming an AR(1) process. Brain activity was convolved over all experimental trials with the canonical hemodynamic response function (HRF) and its derivative.

On the first level, the intra-individual beta contrast weights for three conditions, namely, (1) robot model, (2) human model, and (3) response were evaluated. On the second level, both main effects and individual contrasts were evaluated in a 2 x 2 x 2 flexible factorial design for both the groups (male and female groups) with four conditions, namely, (1) robot model and robotic movements, (2) robot model and anthropomorphic movements (3) human model and anthropomorphic movements, and (4) human model and robotic movements.

For the anatomical localization of effects, the anatomical automatic labeling tool (AAL) in SPM 12 (http://www.cyceron.fr/index.php/en/plateforme-en/freeware) was used to identify Brodmann Areas (BA). Where possible, the SPM Anatomy Toolbox (Eickhoff et al., 2005), available for all published cytoarchitectonic maps from www.fz-juelich.de/ime/spm_anatomy_toolbox, was additionally used and will be indicated in the results by an “Area” specification.

## Results

As a test for the feasibility and the salience of our movement stimuli, we calculated a contrast over all action observation conditions. This contrast [*p* < 0.05 (FWE), *k* = 0] yielded extended activation of the whole visual cortex and the dorsal stream bilaterally (we are presenting the result of this contrast in the Supplementary material).

**Figure 3:**
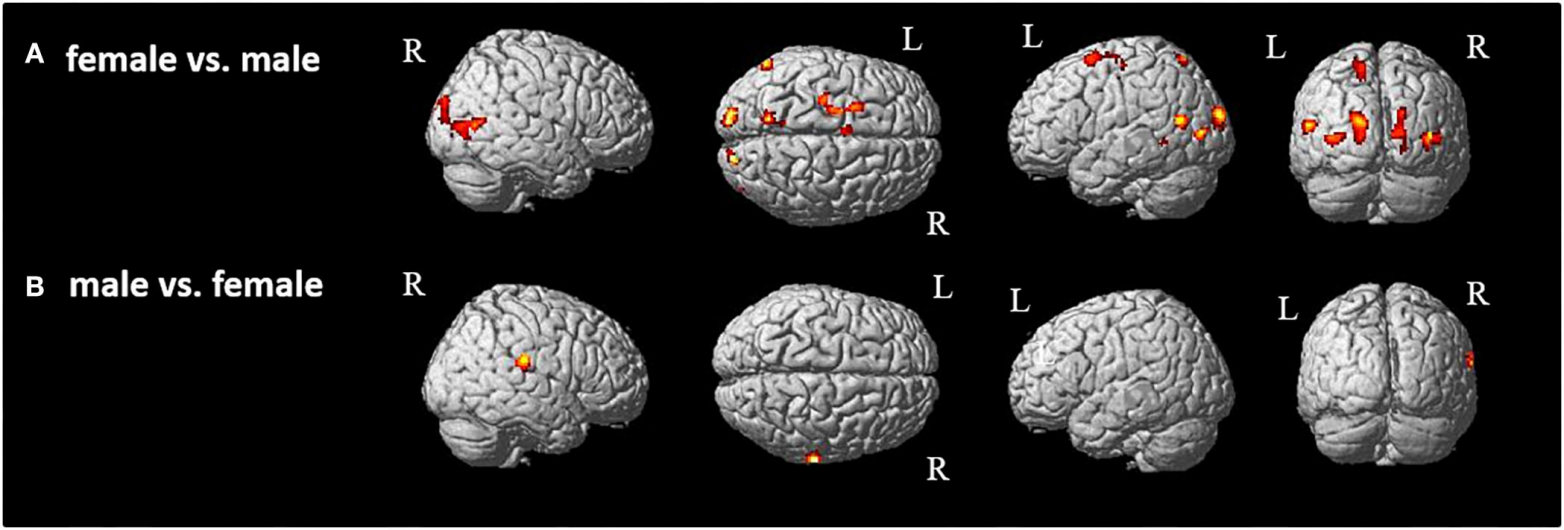
Group comparison between **(A)** female and **(B)** male participants [p < 0.05 (FWE), k = 0]. R, right hemisphere; L, left hemisphere.

Then, as the first step, we performed a main contrast comparing the male and female groups. Therefore, all four conditions (model and movement) were cumulated in the male group and contrasted to all four cumulated conditions in the female group and *vice versa*. As the result, female participants showed significant activations in both hemispheres in primary and secondary visual areas (Figure 3A, Table 1) as well as activations in the left superior parietal and premotor cortex (Figure 3A, Table 1). These activations seem to belong to the dorso-dorsal stream for processing of visual perception of different movements and models in the female group. In contrast, the male group showed significant activation focused on the right hemisphere in the inferior parietal lobe (Figure 3B, Table 1). Here, the activation seems to belong to the ventro-dorsal stream for processing of visual perception of different movements and models by male.

**Table 1:** Significant brain activation of group comparison between female and male participants.

**Contrast**	**Brain region (BA)**	**MNI**			**Cluster size**	**z score**
		**x**	**y**	**z**		
**Female vs. male**	L superior frontal gyrus	−22	6	64	287	5.79
	L precentral gyrus	−30	−20	58		5.58
	L posterior-medial frontal	−6	−2	56	55	5.40
	L postcentral gyrus	−42	−26	38	10	4.78
	[Area 3b]					
	L fusiform gyrus	−40	−50	−10	51	5.27
	[Area FG4]					
	R fusiform gyrus	34	−52	−16	45	5.57
	[Area FG3]					
	L precuneus	−12	−62	54	130	5.22
	[Area 7A (SPL)]					
	L parieto-occipital junction	−57	−66	10	108	6.73
	R middle temporal gyrus	40	−70	13	233	5.44
	[hOc4la]					
	L middle occipital gyrus	−36	−80	6	126	5.86
	[Area hOc4la]					
	R lingual gyrus	20	−80	−2	259	6.20
	[Area hOc3v]					
	R cuneus	16	−92	10		5.40
	[Area hOc1]					
	L superior occipital gyrus	−16	−94	20	256	6.41
	[Area hOc4d]					
**Male vs. female**	R supramarginal gyrus	66	−26	22	108	6.11
	[Area PF (IPL)]					

*L, left hemisphere; R, right hemisphere*.

**Figure 4:**
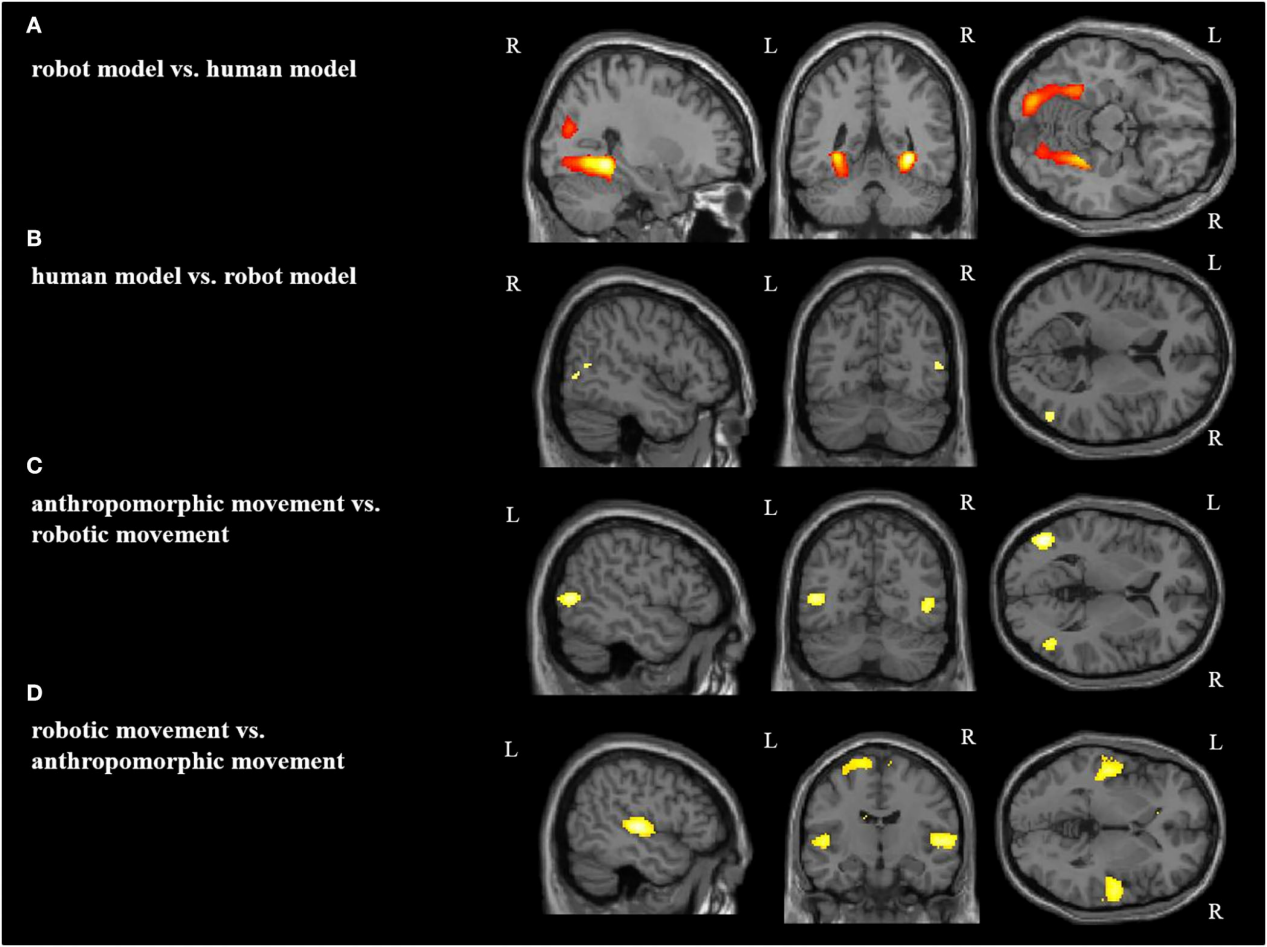
Main effect for each condition: **(A)** robot model vs. human model, **(B)** human model vs. robot model, **(C)** anthropomorphic movement vs. robotic movement, **(D)** robotic movement vs. anthropomorphic movement in the right (R) and left (L) hemisphere [*p* < 0.05 (FWE), *k* = 0].

In the second step, we analyzed the effects of the model (human vs. robot, and *vice versa*) and of the type of movement (anthropomorphic vs. robotic, and *vice versa*) (Figure 4, Table 2). Both the effects of human model and anthropomorphic movement conditions activated the biological movements coding areas in the posterior middle temporal cortex and in the fusiform gyrus (FG). Observation of the robot model showed bilateral activation of the fusiform gyrus, Area FG3, and bilateral in the occipital cortex, whereas observation of the robotic movements activated the temporo-parietal junction, frontal, and primary motor cortices bilaterally.

**Table 2:** Significant brain activation differences for main effect.

**Contrast**	**Brain region (BA)**	**MNI**			**Cluster size**	**z score**
		**x**	**y**	**z**		
Robot model vs. human model	R lingual gyrus	30	−44	−8	1,224	Inf
	[Area hOc3v]					
	L fusiform gyrus	−30	−62	−6	1,441	Inf
	[Area FG3]					
	L lingual gyrus	−30	−52	−6		Inf
	[Area FG3]					
	L lingual Gyrus	−18	−84	−8		Inf
	[Area hOc3v]					
	R middle occipital gyrus	34	−76	20	466	7.56
	L middle occipital gyrus	−32	−92	18	311	7.22
	[Area hOc4lp]					
	L superior occipital gyrus	−10	−100	14	40	5.52
	[Area hOc3d]					
Human model vs. robot model	R fusiform gyrus	42	−44	−18	27	5.48
	[Area FG4]					
	R middle temporal gyrus	50	−64	10	53	4.88
	[Are PGp (IPL)]					
Anthropomrphic movement vs. robotic movement	L fusiform gyrus	−38	−44	−14	2	4.61
	[Area FG3]					
	R middle temporal gyrus	46	−64	2	117	5.56
	[Area hOc5; BA 19]					
	L middle temporal gyrus	−46	−68	6	331	6.87
	[Area hOc4la]					
Robotic movement vs. anthropomorphic movement	R superior medial gyrus	10	36	60	1,372	6.56
	L IFG (p. Triangularis)	−52	42	−2	5	4.59
	[Area 45]					
	L superior medial gyrus	−2	46	30	7	4.59
	R superior temporal gyrus					
	R middle frontal gyrus	30	48	32	474	5.93
	R superior temporal gyrus	54	−14	4	608	6.6
	[Area TE 1.0]					
	L superior temporal gyrus	−50	−20	4	1,942	7.52
	[Area TE 1.0]					
	Thalamus	−20	−32	16		7.09
	R insula lobe	34	−24	10	1	4.53
	[Area lg1]					
	L paracentral lobule	−18	−26	70	2,305	7.04
	[Area 4a]					
	R precentral gyrus	18	−28	72		6.49
	[Area 4a]					
	L precuneus	−6	−44	66		5.26
	[Area 5M (SPL)]					
	cerebellum	0	−58	−12	2	4.61
	cerebellum	0	−62	−10	3	4.52

*L, left hemisphere; R, right hemisphere*.

Third, we wanted to get a better insight into the origin of the observed differences in brain activation in the two sexes.

We started with the calculations of single contrasts between female and male groups for each model (robot and human model) and movement type (anthropomorphic and robotic).

**Figure 5:**
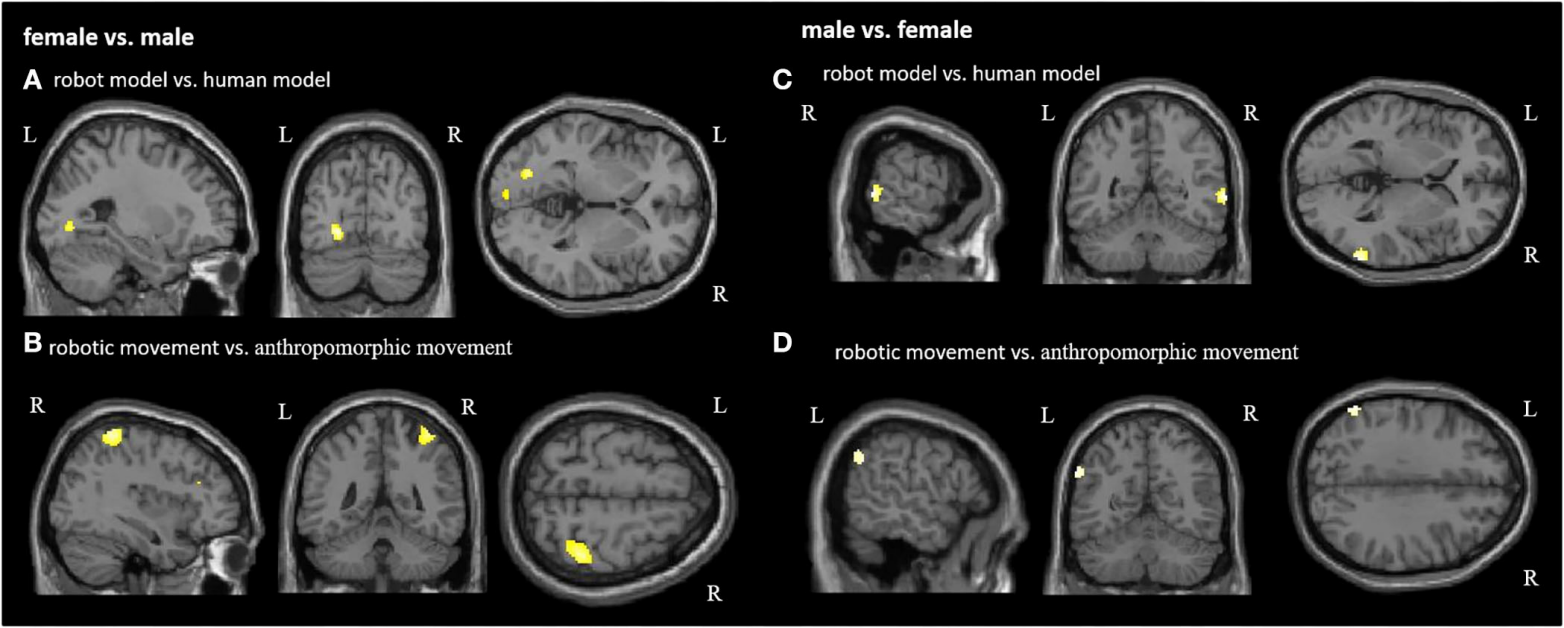
Group comparison between female and male participants in each condition. Significant activations for female participants in the contrast **(A)** robot model vs. human model and **(B)** robotic movement vs. anthropomorphic movement. Significant activations for male participants in the contrast **(C)** robot model vs. human model and **(D)** robotic movement vs. anthropomorphic movement. R, right hemisphere; L, left hemisphere (for visualization purposes, a threshold uncorrected *p* < 0.001, k = 10, was applied).

Figures 5C,D and Table 3 demonstrate the significant activations for the male group (for visualization purposes uncorr. *p* < 0.001*, k* = 10). Observation of the robot model and the robotic movements activated movement coding areas in the posterior parieto-temporal cortex belonging to the ventro-dorsal stream.

**Table 3:** Gender effect for significant brain activation differences for movement (robotic vs. anthropomorphic) and model (robot model vs. human model) comparison.

**Contrast**	**Brain region (BA)**	**MNI**			**Cluster size**	**z score**
		**x**	**y**	**z**		
**Female vs. Male**	R IFG (p. Triangularis)	42	30	26	11	3.37
Robotic movement vs. anthropomorphic movement	L superior frontal gyrus	−30	−4	70	12	3.38
	R postcentral gyurs	42	−42	64	10	4.84
	[Area 1]					
	R superior parietal lobule	30	−74	52	34	3.68
	[Area 7P (SPL)]					
Robot model vs. human model	L lingual gyrus	−22	−74	−4	229	4.63
	[Area hOc4v]					
	L calcarine gyrus	−10	−90	−4		4.42
	[Area hOc1]					
	L superior occipital gyrus	−10	−86	40	11	3.37
	[Area hOc4d]					
	L superior occipital gyrus	−14	−94	30	30	3.71
	[Area hOc4d]					
**Male vs. Female**						
Robotic movement vs. anthropomorphic movement	L Area PGa (IPL)	−60	−58	34	55	3.70
Robot model vs. human model	R middel temporal gyrus	66	−52	2	114	4.14
	[Area PGa (IPL)]					

*L, left hemisphere; R, right hemisphere*.

Figure 5A and Table 3 demonstrate the female activation (for visualization purposes uncorr. *p* < 0.001, *k*=10) for the contrast robot model vs. human model, which contains activation of the left hemispheric primary and secondary visual areas. Regarding the differences between anthropomorphic and robotic movements, female participants showed significant activations (uncorrected *p* < 0.001, k = 10) in the right hemisphere in the primary sensory cortex, the superior parietal lobule, and the visual motor cortex (Figure 5B, Table 3), which is in line with the dorso-dorsal stream.

## Discussion

This study investigated the neural underpinnings of sex differences in the processing of perceived anthropomorphic and robotic movements performed by a digital human model and a gantry robot model. While lying in an fMRI scanner, female and male participants rated the perceived level of anthropomorphism of each of the four model-movement combinations. The behavioral results have been published in the study by Abel et al. (2020): while men were sensitive to differences between robotic and anthropomorphic movements, women attributed equal levels of anthropomorphism to them. Crucially, male participants rated the robotic movements as less anthropomorphic than female participants did, while their rating of the anthropomorphic movements did not differ from the female rating. Neural activity, as assessed by the fMRI investigation, supported our hypothesis of a differential perception of anthropomorphic and robotic movements in female and male individuals. In female participants, overall brain activations (i.e., pooling together anthropomorphic and robotic movements in human and robot models) included the bilateral occipital cortex, the left parieto-occipital junction, the left superior parietal cortex, and the left dorsal premotor cortex. Differently, in male participants, overall activations involved the right supramarginal gyrus. We would like to discuss these results in the light of the newly proposed subdivision of the dorsal stream of visual information processing and the results of the accompanying behavioral assessment in the study by Abel et al. (2020).

As already mentioned in the “Introduction” section, the subdivision of the visual information processing in divided into two parallel pathways, namely, the dorsal “where” or “how” stream, and the ventral “what” stream (Ungerleider and Mishkin, 1982; Goodale and Milner, 1992) was recently refined. Indeed, a further subdivision of the dorsal stream into a dorso-dorsal and a ventro-dorsal stream has been proposed (Rizzolatti and Matelli, 2003; Binkofski and Fink, 2005; Pisella et al., 2006; Binkofski and Buxbaum, 2013). The dorso-dorsal stream is originating in the primary visual cortex and goes through the superior parietal and dorsal premotor cortex, whereas the ventro-dorsal stream goes through inferior parietal and ventral premotor cortex. The dorso-dorsal stream is responsible for online motor control with little working memory capacity; instead, the ventro-dorsal stream is equipped with more working memory capacity and is responsible for memory-driven motor control and motor simulation (Rizzolatti and Matelli, 2003; Binkofski and Buxbaum, 2013). In female participants, the above reported pattern of brain activations belongs to the dorso-dorsal stream (Rizzolatti and Matelli, 2003; Binkofski and Fink, 2005; Pisella et al., 2006; Buxbaum and Kalenine, 2010; Binkofski and Buxbaum, 2013; Binkofski and Buccino, 2018). In male participants, instead, overall activations are attributable to the ventro-dorsal stream (Rizzolatti and Matelli, 2003; Binkofski and Fink, 2005; Pisella et al., 2006; Binkofski and Buxbaum, 2013; Binkofski and Buccino, 2018). Such differential activation of the two dorsal sub-streams in the two sex groups shed some light on the peculiar mode of overall movement processing in human females and males. Indeed, men would rely on previous knowledge about different types of movements, whereas women would tend to analyze these movements online. These different overall patterns of activations in the two groups would explain, to some extent, the behavioral differences observed in the study by Abel et al. (2020) for movements rating. Indeed, the female attitude to favor online processing of perceived movements (i.e., dorso-dorsal stream) would translate into assigning similar anthropomorphic features to robotic movements as compared to anthropomorphic ones. Conversely, the male “preference” for the processing of perceived movements based on previous knowledge (i.e., ventro-dorsal stream) would be consistent with their higher sensitivity to differences between robotic and anthropomorphic movements, and to their judgment of the robotic movements as less anthropomorphic than judgment of women.

For the sake of completeness, our MRI results regarding contrasts over all action observation conditions show activation in the whole visual cortex. This finding underpins the salience of our stimuli for activation of the motion coding areas. Further results show activations in the biological movement coding areas for human model and anthropomorphic movement conditions. These results highlight that participants distinguish between biological and non-biological movements. The observation of the robot model shows bilateral activations of the fusiform gyrus, Area FG3, and bilateral in the occipital cortex. This suggests that participants required higher effort and attention to identify and analyze the robot model (Weiner and Zilles, 2016). Interestingly, Area FG3 has been associated with the perception of scenes, buildings, and places, whereas Area FG4 is associated with the perception of body parts and faces (Lorenz et al., 2017). This implies that the participants identify the robot model in a more abstract way, while the human model is characterized by biological markers. Observation of the robotic movements activated the temporo-parietal junction, frontal, and primary motor cortices bilaterally, which indicated that they were associated with the analysis of complex movements (Schultz et al., 2004). Regarding sex differences in the perception of the models, male participants show stronger activation of the right IPL in the robot model condition, which has a role in the analytical perception of movements (Rizzolatti et al., 2006). In contrast, female participants show stronger activations in the primary visual areas of the left hemisphere (V3 and V1), which are related to the perception of movements, in general.

In sum, our findings suggest that female and male individuals differ for what concerns attitudes in movements processing, as well as in the brain areas that process the appearance of robots.

Transferred to everyday life, such as healthcare or rehabilitation, as well as industrial environment, it should be considered that male and female subjects process movements differently, and that such crucial factor should be accounted in the evaluation of the feelings of trust transmitted by a robot assistant. Further research is therefore needed to determine which feelings of different robots regarding the perceived movements trigger in people, in order to create a working atmosphere that is as relaxed and trusting as possible.

## Conclusion, limitation, and perspectives

This study investigated biological sex differences for the processing of perceived robotic and anthropomorphic movements in the human brain. Our results suggest that female subjects tend to use an online mode of action processing conveyed by their dorso-dorsal stream, whereas male subjects use more knowledge-based analysis as processed by the ventro-dorsal stream. Indeed, these results represent one first brain imaging evidence of sex differences in the perception of movements. Our results can have crucial implications in research domains like the acceptance of robotic systems in the manufacturing environment. Crucially, the implementation of humanoid service, or co-workers robots in modern industrial production, healthcare industry, or rehabilitation, could be “tailored” to the sex of the user for what concerns their movement features.

Regarding the possible limitations of this study, one might argue that if, on the one hand, a brain imaging facility represents the golden standard to identify patterns of functional activations in the brain; on the other hand, to have the participant lying in the MRI-scanner watching video clips of moving robots is quite far from providing a realistic interactive context. It would be interesting to evaluate our same experimental paradigm in a real working environment with the utilization of portable functional near-infrared spectroscopy (fNIRS) technology. Finally, this study deserves a follow-up investigation for what concerns the emotional processes in male and female participants interacting with different robots.

## Data availability statement

The raw data supporting the conclusions of this article will be made available by the authors, without undue reservation.

## Ethics statement

The studies involving human participants were reviewed and approved by Institutional Ethics Review Board of the Medical Faculty at RWTH Aachen University (EK 2013/14). The patients/participants provided their written informed consent to participate in this study.

## Author contributions

MA: planning and implementation of the study, evaluation and interpretation of the data, and writing and revision of the manuscript. SK and HP: planning and implementation of the study and evaluation of the video material. HJP: implementation of the study and evaluation of the data. JK and CS: supervision of the study. AS: evaluation of the data. AP: evaluation of the data, discussion of the results, and revision of the manuscript. FB: conception and supervision the study, discussion of the results, and revision of the manuscript.

## Funding

This study was supported by the Federal Ministry of Education and Research (BMBF) under Grant No. 16SV7013.

## Conflict of interest

The authors declare that the research was conducted in the absence of any commercial or financial relationships that could be construed as a potential conflict of interest.

## Publisher's note

All claims expressed in this article are solely those of the authors and do not necessarily represent those of their affiliated organizations, or those of the publisher, the editors and the reviewers. Any product that may be evaluated in this article, or claim that may be made by its manufacturer, is not guaranteed or endorsed by the publisher.

## Footnotes

^1^We recall here the distinction between *gender* and *sex*: “Gender refers to psychological, social and cultural factors that shape attitudes, behaviors, stereotypes, technologies and knowledge,” whereas “Sex refers to the biological attributes that distinguish organisms as male, female, intersex” (Tannenbaum et al., 2019). In this study, we investigated biological sex differences in the human brain.
