# Supplementary material for: Anthropomorphic or non-anthropomorphic? Effects of biological sex in observation of actions in a digital human model and a gantry robot model
Source: Front Neurorobot. 2022 Aug 17;16:937452. doi: 10.3389/fnbot.2022.937452 (PMC9428556; doi:10.3389/fnbot.2022.937452)
Supplement: Supplementary file 1 [file Data_Sheet_1.pdf]

Supplementary Material

Table 1: Activation related to action observation in all four conditions. L: left hemisphere, R: right hemisphere.

Brain region (BA)	MNI			Cluster size	z score
	x	y	z		
L prefrontal cortex (PFC) [BA10]	-36	50	20	43	5.14
R orbitofrontal cortex [Area Fo3]	22	40	-12	44	7.01
R prefrontal cortex (PFC) [Area Fp1; BA10]	20	54	-12		4.70
R middle temporal gyrus [Area hOc5; BA19]	46	-66	4	15352	Inf.
R fusiform gyrus [Area FG4]	40	-52	-12		Inf.
R visual cortex [Area hOc3v]	22	-78	-8		Inf.
L middle temporal gyrus [Area hOc5; BA19]	-44	-70	4	30868	Inf.
L fusiform gyrus [Area FG4]	-38	-54	-14		Inf.

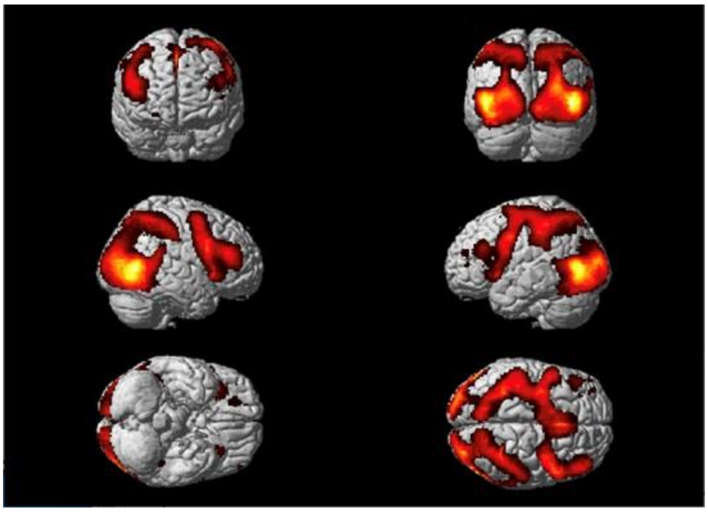

Figure 1: Activation related to action observation in all four conditions (*p*<.05 (FWE), *k*=0).
